# A Retrospective Cohort Study of Clinical Factors Associated with Transitions of Care among COVID-19 Patients

**DOI:** 10.3390/jcm10194605

**Published:** 2021-10-08

**Authors:** Dennis Swearingen, Gregory Boverman, Kristen Tgavalekos, David P. Noren, Shreyas Ravindranath, Erina Ghosh, Minnan Xu, Lisa Wondrely, Pam Thompson, J. David Cowden, Corneliu Antonescu

**Affiliations:** 1 Department of Medical Informatics, Banner Health, Phoenix, AZ 85012, USA; dennis.swearingen@bannerhealth.com (D.S.); Pam.Thompson@bannerhealth.com (P.T.); jdavid.cowden@bannerhealth.com (J.D.C.); corneliu.antonescu@bannerhealth.com (C.A.); 2 Department of Biomedical Informatics, University of Arizona College of Medicine, Phoenix, AZ 85004, USA; 3 Connected Care and Personal Health Department, Philips Research North America, Cambridge, MA 02141, USA; kristen.tgavalekos@philips.com (K.T.); david.noren@philips.com (D.P.N.); shreyas.ravindranath@philips.com (S.R.); erina.ghosh@philips.com (E.G.); Minnan.Xu@philips.com (M.X.); lisa.wondrely@philips.com (L.W.)

**Keywords:** COVID-19, triage, care transitions

## Abstract

Coronavirus Disease 2019 (COVID-19) is an international health crisis. In this article, we report on patient characteristics associated with care transitions of: 1) hospital admission from the emergency department (ED) and 2) escalation to the intensive care unit (ICU). Analysis of data from the electronic medical record (EMR) was performed for patients with COVID-19 seen in the ED of a large Western U.S. Health System from April to August of 2020, totaling 10,079 encounters. Of these, 5172 resulted in admission as an inpatient within 72 h. Inpatient encounters (*n* = 6079) were also considered for patients with positive COVID-19 test results, of which 970 resulted in a transfer to the ICU or in-hospital mortality. Laboratory results, vital signs, symptoms, and comorbidities were investigated for each of these care transitions. Different top risk factors were found, but two factors common to hospital admission and ICU transfer were respiratory rate and the need for oxygen support. Comorbidities common to both settings were cerebrovascular disease and congestive heart failure. Regarding laboratory results, the neutrophil-to-lymphocyte ratio was associated with transitions to higher levels of care, along with the ratio of aspartate aminotransferase (AST) to alanine aminotransferase (ALT).

## 1. Introduction

The Coronavirus Disease 2019 (COVID-19) pandemic began in December 2019, with the first case reported in the United States in January 2020. As of 22 June 2021, there were 178 million confirmed cases and 3.8 million deaths reported globally [1]. The pandemic has severely strained hospital operations and care delivery. In response, hospitals have put in place a range of strategies to address such challenges across care settings. For example, emergency departments (EDs) have adapted or created new practices to address the surge of patients who potentially present with COVID-19. In a primer for emergency physicians, it was advised that patients with mild symptoms, no significant comorbidities, and the ability to self-quarantine should be discharged home [2]. Furthermore, predictors of outcomes can be used to support the clinical management of patients and to inform resource allocation decisions. At George Washington University, a clinical decision pathway for emergency department (ED) triaging was introduced to protect ED bed capacity [3]. Among the pathway components was a labeling of patients as high risk or low risk according to age, heart rate, and temperature criteria.

In one study of patients hospitalized for COVID-19, approximately 30% required ICU care [4]. A key element of preparation for patient surges is a plan for increasing critical care capacity as needed, for example by expanding to other units like post-anesthesia and step-down units [5]. Given the challenges associated with capacity, resource management, staffing, and triage [6], predictors of the need for a patient to be transitioned to the ICU could be quite valuable.

The characteristics, comorbidities, and outcomes of COVID-19 patients have been studied in various regions around the world – Wuhan, China [7], the Lombardy region of Italy [8], and the New York City area in the United States [9], to name a few. The objective of this study is to identify factors associated with care transitions for a large cohort of COVID-19 patients within an integrated regional health system. The hypothesis is that several clinical measurements and conditions would be identifiable as associated with the transfer of these patients from the ED to the inpatient floor and also to the ICU.

## 2. Materials and Methods 

Electronic medical record (EMR) data was retrospectively obtained and de-identified to a limited dataset, for patients seen in a large U.S. health system. This regional system is broad-based, consisting of approximately 30 acute-care hospitals which range from community to tertiary-care facilities located in the states of Arizona, California, Colorado, Nebraska, Nevada, and Wyoming. All cases across locations were aggregated and variations in practice or procedure between different facilities were not explored. Patients presenting to the facilities between 1 April and 30 July 2020 were considered for inclusion and a diagnosis of COVID-19 was identified based on the assignment of ICD10 code U07.1.

To validate that the patient transitions extracted from the EMR made sense, a network graph was created to illustrate transitions through the health system. Nodes were used to represent location types or discharge dispositions. Miscellaneous location types with low counts (e.g., obstetrics ward, behavioral health ward, etc.) were grouped together. Also, miscellaneous discharge dispositions with low counts (e.g., discharge to hospice, discharge to jail, left against medical advice, etc.) were grouped together. For this reason, and also because a single patient may have experienced a transition multiple times, the sum of the edges originating from a node was not intended to add up exactly to the number in the node. Rather, the numbers indicated the general proportion of encounters in each location.

The patients were divided into two groups: those who presented to the ED and those who were admitted to the hospital from a non-ED setting. The first group was further divided into four cohorts based on disposition at ED discharge. Cohort A consisted of patients who were discharged to home and were not admitted as an inpatient within 72 h. Cohort B consisted of patients who were discharged to home but were admitted as an inpatient within 72 h. Cohort C were patients who were admitted to the hospital at the end of their ED visit. The remaining patients, consisting of patients who were transferred to another facility or left against medical advice, were excluded from the analysis. Among the patients who were directly admitted to the hospital, patients who were directly admitted to the ICU were excluded from the analysis. Inpatient deterioration was defined as requiring transfer to the ICU or experiencing in-hospital mortality. Among all patients admitted to a non-ICU unit, the patients with a missing discharge disposition were excluded. The remaining inpatients were subdivided into two cohorts: Cohort D consisted of inpatients who did not deteriorate and Cohort E consisted of inpatients who did deteriorate. Table 1 provides a summary of cohort descriptions and their endpoints.

Demographic factors (age, race, ethnicity, sex) are reported for all cohorts. For summarizing demographics, we used the tableone package for Python 3.0 (Wilmington, DE, USA) [10,11]. Continuous variables were summarized as a mean with standard deviation (SD) in parentheses. Categorical variables were summarized as counts with the percentage of their respective classes in parentheses. For categorical variables with at least 5 counts per category and class, a Chi-squared test was performed to test the null hypothesis that there is no difference in the frequencies for each category between the two classes. For continuous variables, a two-sample *t*-test was performed to test the null hypothesis that the means of the two populations were equal. *p*-values describe the probability of observing results at least as extreme under the assumption that the null hypothesis is correct. A *p*-value less than 0.05 was used as the significance level below which the null hypothesis was rejected.

Charlson comorbidities were specified as defined in Quan et al. 2011 [12] and were calculated from patient diagnosis codes (ICD10) using the ICD package in the R statistical software [13]. Note that patient diagnosis codes were charted based on treatment during the visit and may not have reflected the patient’s full history of chronic disease. Three sets of cohort comparisons were performed, and they are described in Table 2 along with the time range over which data was considered.

Comparisons 1, 2, and 3 each include an outcome linked with higher acuity (ED admission, ED bounce-back, or inpatient deterioration, respectively) that is compared to an outcome of lower acuity. To study the higher acuity outcomes, univariate logistic regression models were built for different variables-comorbidities, symptoms, vital signs, and lab results. All non-categorical variables were standardized to have zero mean and unit variance before model generation. For those covariates deemed to be highly statistically significant (*p*-value less than 0.001), we ordered them by the absolute value of the logistic regression coefficient, showing the top 30 covariates for each outcome (or fewer, if there were not 30 that were significant).

Multivariate results were generated using stepwise regression with a forward-backward procedure where covariates were included if their *p*-values in the multivariate fit were less than a given threshold (0.01). Likewise, covariates were excluded if their *p*-values in the multivariate fit exceeded a given threshold (0.05).

This project was a part of an ongoing retrospective deterioration detection study approved by the Institutional Review Board of the health system. The study was not registered with ClinicalTrials.gov because it is non-interventional (observational) clinical research and does not meet the definition of an applicable clinical trial. 

## 3. Results

**Figure 1:**
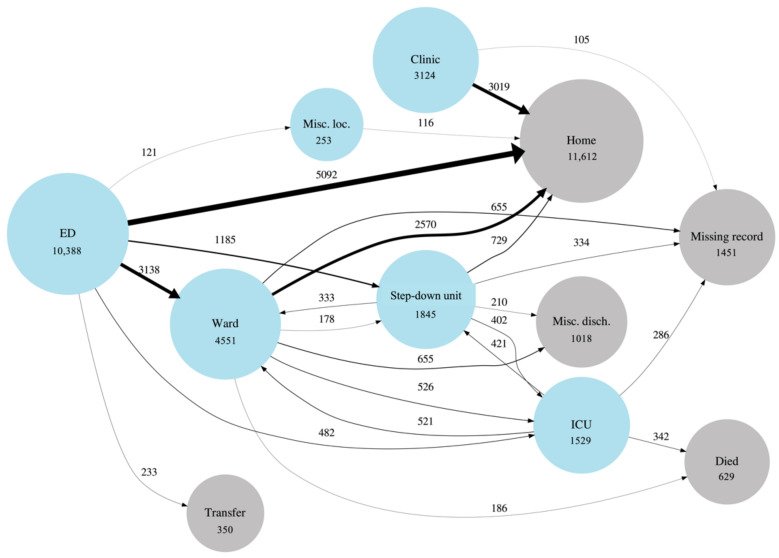
Graphical depiction of COVID patient flow through the healthcare system. Blue circles (nodes) represent location types within the system. Grey circles represent discharge dispositions. Circle diameters are proportional to the (natural log of the) number of patients who passed through a location type at least once during an encounter. Line (edge) widths are linearly proportional to the number of times a transition occurred and the edges are also labeled with this number. The “Misc. loc.” node stands for miscellaneous locations that have been grouped together due to low counts and for privacy purposes. The “Misc. disch.” node stands for miscellaneous discharge dispositions that have been grouped together. For visualization and privacy purposes, transitions are only shown when they have more than 100 counts.

Figure 1 is a network graph that illustrates patient transitions through the health system. Patient discharge from the ED to home was the most prevalent transition type (51%). Of the ED patients who were admitted to the hospital, approximately 32% were placed in a low acuity general ward first, while approximately 17% were admitted to a higher acuity setting (ICU or Step). Note that in-hospital death occurred mostly in the ICU setting.

**Figure 2:**
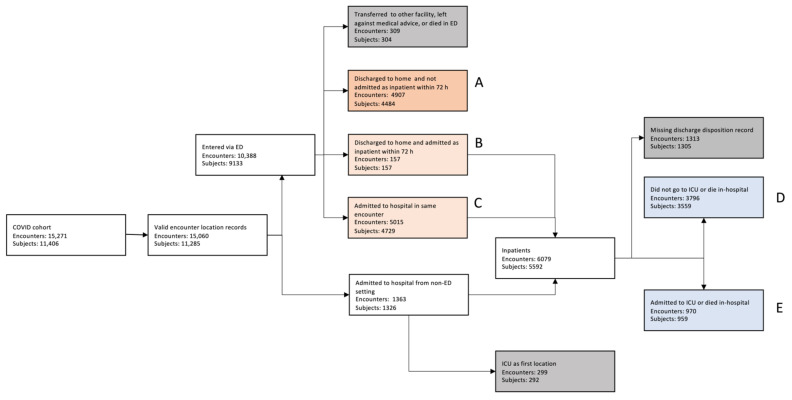
CONSORT diagram for patients included in the study.

We identified 11,406 unique patients/subjects with the COVID-19 diagnosis who had 15,271 unique encounters with the hospital system within the time range considered. Figure 2 shows the CONSORT diagram for patients included in the study. See Table A1 and Table A2 for cohort demographics.

### 3.1. Emergency Department (ED) Cohort

The following Section 3.1.1 and Section 3.1.2, report results for Comparisons 1 (ED admission) and 2 (ED bounce-back), as described in Table 2. These investigate the factors associated with transitions of care from the ED.

#### 3.1.1. Comorbidities and ED Transitions

**Figure A1:**
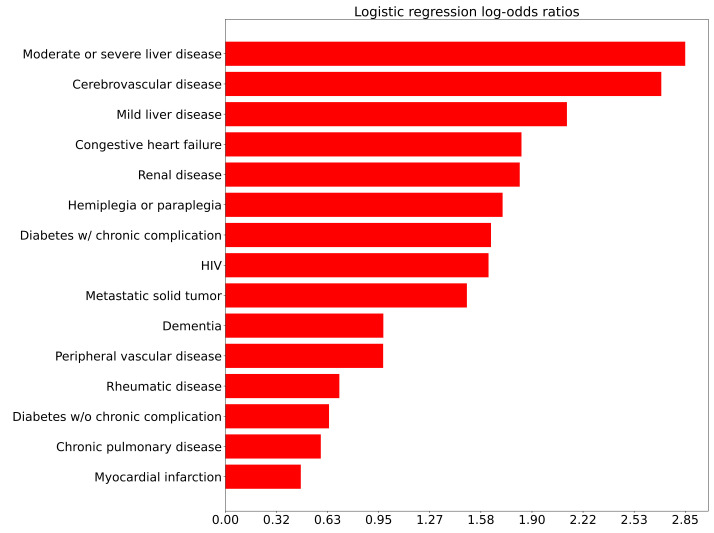
Logistic regression log odds ratios of comorbidities for Comparison 1 (ED admission) adjusted for age.

**Figure A2:**
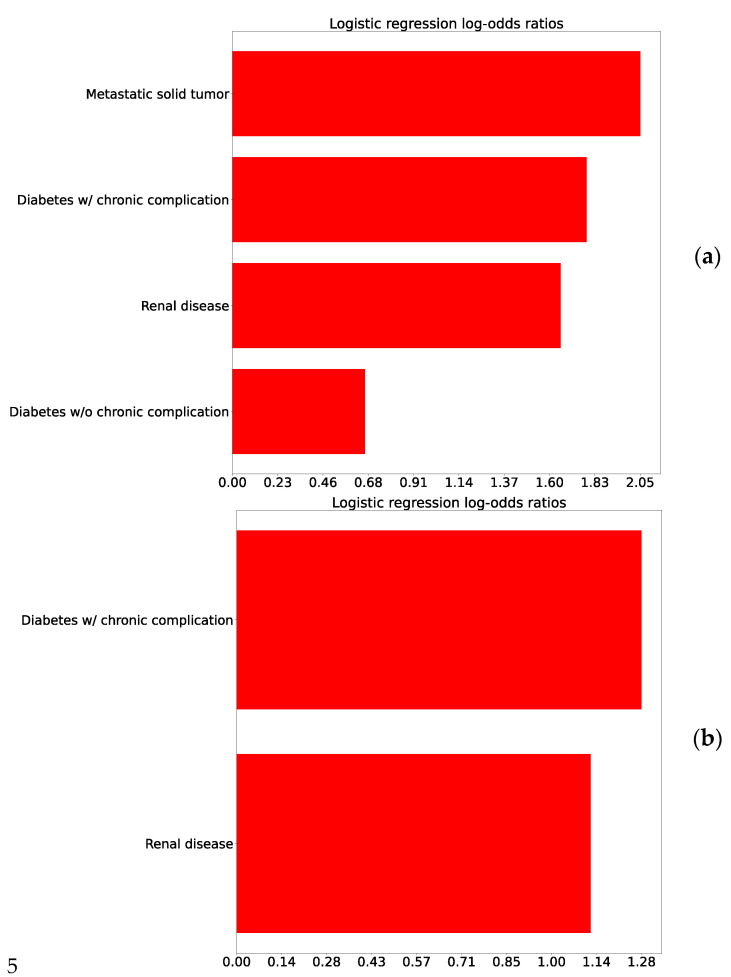
Logistic regression log-odds ratio correlating comorbidities to the outcome for comparison 2: (**a**) Raw log odds ratios and (**b**) Controlled for age.

Logistic regression log-odds ratios correlating comorbidities to the outcomes of interest for Comparison 1 (ED admission) are shown in Figure A1. Only statistically significant correlations (*p* < 0.01) are displayed and they are adjusted for age. Results for Comparison 2 (ED bounce-back) are shown in the Appendix A in Figure A2.

#### 3.1.2. Correlates of Vital Signs, Symptoms, and Laboratory Results to ED Transitions

**Figure 3:**
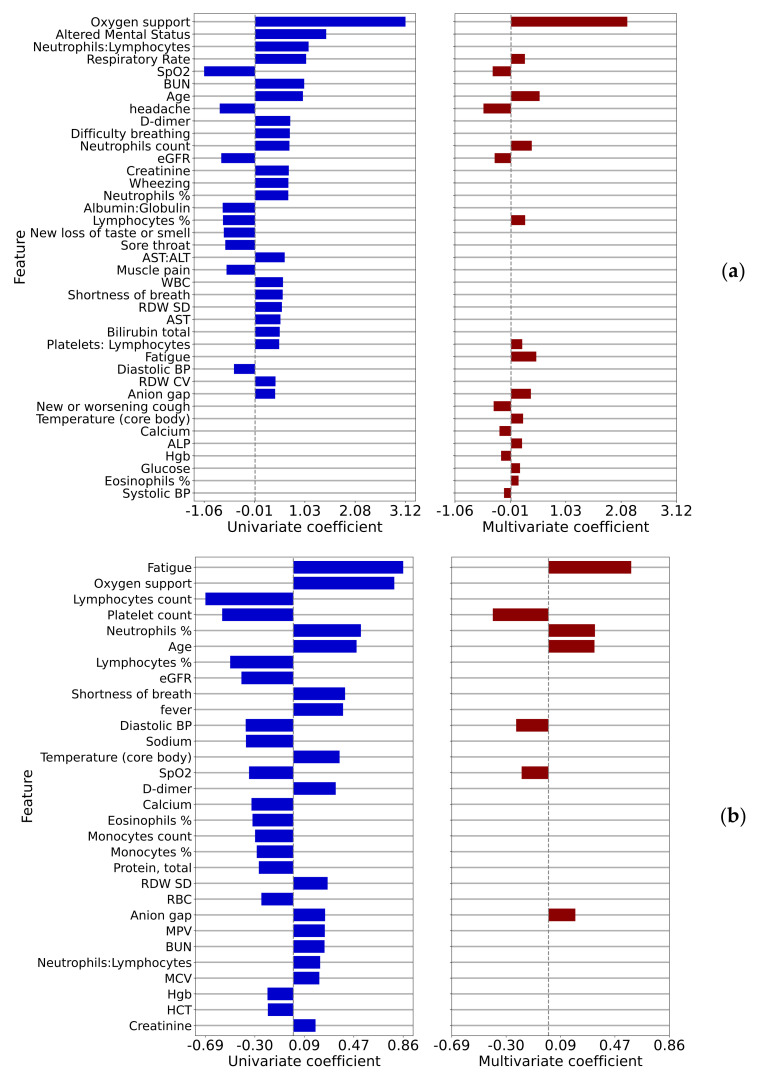
Univariate and multivariate logistic regression coefficients for comparisons 1 (**a**) ED admission and 2 (**b**) ED bounce-back.

Logistic regression log odds coefficients for the outcomes of interest are shown in Figure 3, where Comparison 1 (ED admission) is shown in (a), and Comparison 2 (ED bounce-back) is shown in (b). As previously, only statistically significant correlations (*p* < 0.01) are shown. We show univariate and multivariate coefficients in the same figure with no values shown for parameters that did not appear in the multivariate model. Here the variables are sorted by the maximum of the absolute values of the univariate and multivariate coefficients (this applies to all figures of this type that follow). Quantitative multivariate results for comparisons 1 and 2 are shown in Table A3 and Table A4.

### 3.2. Non-Critical Inpatient Cohort

The following Section 3.2.1 and Section 3.2.2, report results for Comparison 3 (inpatient deterioration).

#### 3.2.1. Correlations of Comorbidities with Inpatient Deterioration

**Figure A3:**
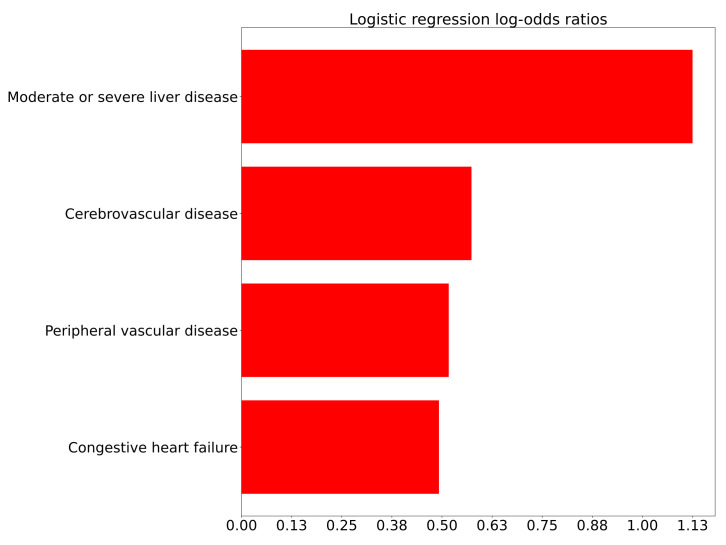
Logistic log-odds coefficients correlating comorbidities to the outcome for Comparison 3 (inpatient deterioration), controlling for age.

The correlations of comorbidities to the outcome for Comparison 3 (inpatient deterioration) are shown in Figure A3, adjusted for age. Of note, relatively few comorbidities were correlated to the outcome with statistical significance and the coefficients for those that were significant were smaller than for Comparison 1 (ED admission).

#### 3.2.2. Correlations of Symptoms, Vital Signs, and Laboratory Results with Inpatient Deterioration

**Figure 4:**
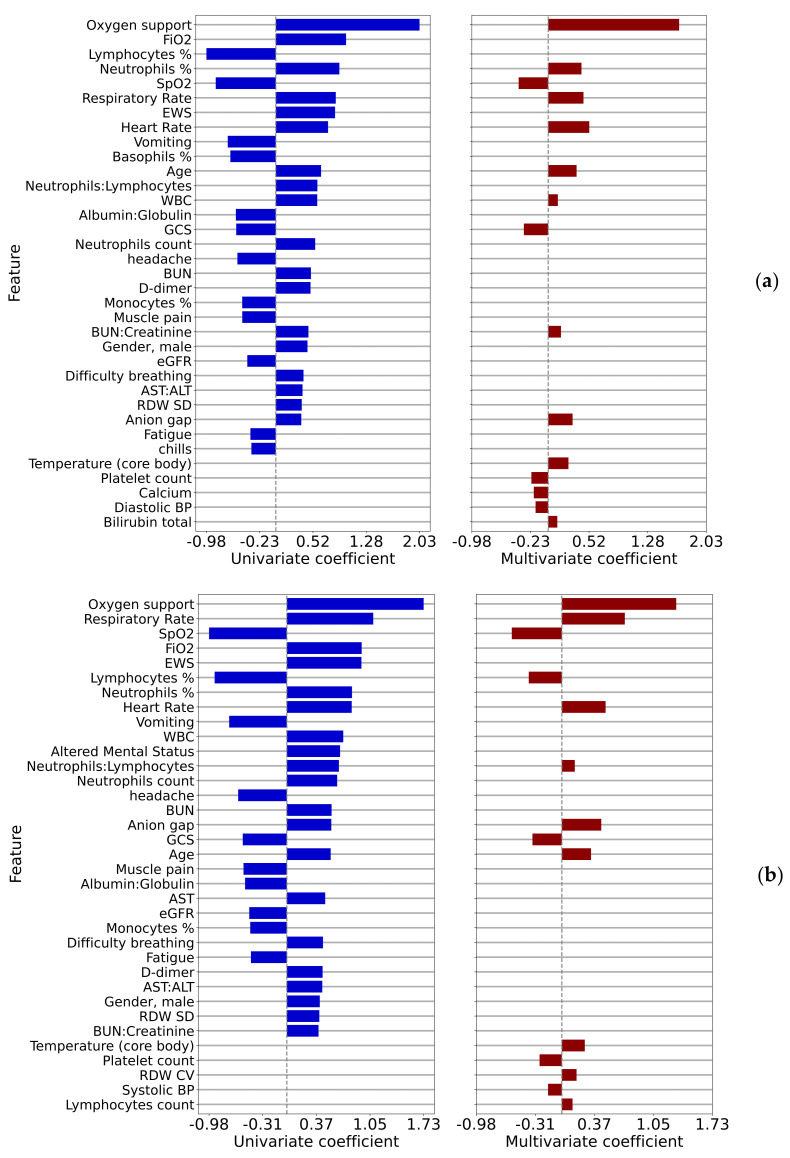
Univariate and multivariate regression coefficients for comparison 3 (**a**) 12 h prior to the event and (**b**) 4 h prior to the event.

The univariate and multivariate logistic regression log-odds ratios correlating measured parameters to the outcome of patient deterioration in 12 h are shown in Figure 4a and to the outcome of patient deterioration in 4 h are shown in Figure 4b, where only statistically significant correlations (*p* < 0.01) are displayed.

Vital signs, notably heart rate and respiratory rate, are among the strongest correlates. In the 12-h case, the percentage of neutrophils is a sign of imminent deterioration. The common variables to all four multivariate models were SpO2, anion gap, age, neutrophil-related variables (count, %, or ratio to lymphocytes), platelet-related variables (count or ratio to lymphocytes), and blood pressure (systolic or diastolic). Quantitative results for these two-time points are shown in Table A5 for the 12-h case and in Table A6 for the 4-h case

## 4. Discussion

The COVID-19 pandemic forced hospital systems and care providers to evaluate and treat patients while under significant resource constraints. Part of the challenge in doing so was determining the appropriate care transitions for these patients, including whether to admit patients from the ED to the floor or to transfer from the floor to the ICU. Each of these transition decisions requires an assessment of patient acuity, which was challenging given the sparsity of information about COVID-19, particularly early in the pandemic.

The selected date range for this study is 1 April through 30 July 2020. It was chosen to reduce bias or inconsistency from factors such as changes in clinical standards, overall bed management, and therapeutic interventions. These changes were seen, sometimes to a significant extent, as the pandemic moved through waves and also as understanding increased. By focusing on the first wave of a particular health system’s geography, greater clarity may be present from the data.

We have developed four multivariate models correlating measured patient covariates with the outcomes described in Table 2. The summary results for these models can be found in Table A7, where we have computed the rank of each covariate in each of the models and counted the number of models in which that covariate appears. The analysis of these summary results tells us which covariates are correlated to outcomes across all care settings vs. those covariates which are specific to settings. We found that SpO2, age, and anion gap appeared with high statistical significance and relatively high ranks in all of the models. Of these, perhaps only anion gap is surprising, possibly indicating the risk of ketoacidosis in diabetic patients. Oxygen support, platelet count, core body temperature, and respiratory rate appeared in three of the four models. We further note that percentages of lymphocytes or neutrophils (which are in some sense complementary as these are the most common white blood cells) appear in all four of the models. Similarly, systolic or diastolic blood pressure, which are also complementary, appear in all four of the models as well. Finally, some covariates appear more important in some care settings vs. others. For example, self-reported fatigue appears to be very important to the decision to admit as an inpatient (given its high ranking as shown in Figure 3b) but less important for ICU transfer. Likewise, heart rate is very important to the decision to admit a patient to the ICU but less important in the initial admission decision from the ED.

Univariate analysis of comorbidities and complications related to admission as inpatient from the ED is presented in Figure A1. It may be interesting to note that cardiovascular comorbidities such as cerebrovascular disease, congestive heart failure, and peripheral vascular disease have higher log-odds ratios as compared to pulmonary comorbidities. With the early focus of COVID-19 as a pulmonary-oriented infection and the need for respiratory support, some readers may find this unexpected. However, this finding is similar to a recently-published multivariate analysis which showed that asthma, chronic obstructive pulmonary disease, and use of corticosteroid treatment were not independent risk factors for the increased acuity outcomes of ICU admission or death [14]. On the other hand, cardiovascular comorbidities are known to be frequently present among COVID-19 patients and may be associated with adverse clinical events and mortality [15,16]. Among clinical characteristics, oxygen support had by far the highest correlation with admission as an inpatient, with the greatest inverse correlation being SpO2. These two findings are perhaps expected and are indicative of the effects of COVID-19 on the respiratory system, even in patients without known pulmonary comorbidities or complications.

A notable item with univariate log-odds ratios associated with inpatient admission is d-dimer, which is among the top biomarkers associated with COVID-19 severity [17,18,19]. A less-expected association that arose was red blood cell distribution width (RDW). However, RDW is recognized as a biomarker for all-cause mortality and several studies have now identified increased RDW as a biomarker for COVID-19 severity as well [20,21,22]. Another interesting association with acuity was aspartate aminotransferase (AST)-dominant aminotransferase elevation, which has been reported in the literature [23]. Furthermore, the presence of certain symptoms such as headache, sore throat, and muscle pain are inversely associated with admission; perhaps this could be explained by the concept that patients with non-COVID infectious, or non-infectious, conditions that typically do not require admission may frequently present with these symptoms.

One potentially interesting and novel finding is the relatively strong association of moderate or severe liver disease to admission from the ED (Figure A1) and inpatient deterioration (Figure A3). These comorbidities are not generally believed to be associated with COVID-19 and a potentially confounding factor is obesity. Perhaps there are mechanisms related to contributing conditions such as fatty liver disease, alcoholic disease, NASH (non-alcoholic steatohepatitis), or viral hepatitis, which might be independent risk factors in and of themselves.

Of interest for further investigation are the associations of higher % neutrophils and lower % lymphocytes with higher acuity, which were found also in other publications [24,25]. Potential causes for this finding of relative lymphopenia are the inflammatory cytokine storm, exhaustion of T cells, infection of T cells, and interference with T cell expansion [26]. Zhu et al. note that in addition to a high neutrophil to lymphocyte ratio, a low ratio may also be associated with mortality, indicating a nonlinear association with acuity [27].

Addressing the concept of ED bounce-backs, studied in Comparison 2, can be quite useful for a health system. Many resources and clinical outcomes may be impacted by patients who were sent home but then presented within 72 h and resulted in hospital admission. Attention to the symptoms, vitals, and lab results that correlate in a positive or negative risk for this phenomenon could not only save time and expense for both the health system and patients, but also lead to potential improvement in clinical outcomes. Figure 3b that the top three risks include the symptom of fatigue, the need for nasal canula oxygen support, and the increase in the percent neutrophils value. On the other side of the log-odds ratios were found decreased percentage and number of lymphocytes, platelet count, and eGFR.

A patient who suffers deterioration rather acutely, within 4–12 h, is an important situation to identify within a hospital. Various risk models and algorithms have been developed in this space for clinical conditions such as sepsis. Here we look at the comorbidities (Figure A3) and the covariates such as lab values (Figure 4) for inpatient deterioration. Factors associated with ED to inpatient transition are mostly consistent with the factors associated with ICU transfer or in-hospital mortality. A recent study has found that COVID-19 patients with dementia have a higher rate of hospitalization and mortality compared to COVID-19 patients without dementia. It includes potential reasons for this finding [28] but this study did not find dementia to be associated with patient deterioration after adjusting for age, in contrast to the ED admission case, where a positive association was found. Studying Figure 4b, the analysis of deterioration within 4 h, shows the leading risks to be the need for oxygen support and respiratory rate. These are perhaps rather expected; however, still relatively high on the list is an altered mental status. This finding may point to the need for an additional risk analysis by the treating physician based upon pre-existing, and developing, mental faculty conditions that may not be typically brought to attention as much as pulmonary and cardiovascular review.

Comparing the covariates associated with patient deterioration at 4 and 12 h, shown in Figure 4a,b, we see very similar patterns, though some subtle differences, for example the logistic regression log-odds ratio for the respiratory rate increasing from 0.45 to 0.72 between these time points.

This work had some limitations. Unstructured data (e.g., progress notes, readings of CT scans) were not incorporated into the analysis. This type of data might clarify other factors related to the decision to admit. Also, regarding ED bounce-backs and inpatient transition to higher acuity, we did not try to quantify the patient’s clinical stage of presentation with a systematic scoring algorithm, which may have revealed contributing factors to disease trajectory. Finally, for the multivariate analyses, it was found that the exact variables included in the fits were dependent on the “in” and “out” parameters for the stepwise regression procedure and therefore the results are dependent on the specifics of the analysis method.

## 5. Conclusions

Confirmation of the hypothesis was achieved; several clinical measurements and conditions were able to be identified as associated with care transitions among COVID-19 patients. During the investigation, this study produced a baseline analysis of clinical covariates and comorbidities with respect to organizing and statistically ranking, the findings related to both positive and negative risk. Some of the same risk factors were present for the transition from ED to inpatient as from inpatient to deterioration (ICU or death). In addition, sub-analyses looking at bounce-back admissions were also presented for both positive and negative risk. Furthermore, a multivariate analysis of the transitions and sub-analyses also revealed a few similarities and differences relative to univariate.

The logistic regression log-odd ratios data presented in this work deserve further contemplation and review. It could promote further investigation, as some findings may be either confirmatory or unexpected regarding relative strength or direction. We look forward to advancements in understanding of how the presence of particular comorbidities and covariates such as lab values, can help guide the care and resource utilization related to COVID-19 patients. Future work by this team may include further analysis of demographic factors which influence transitions of care, a generalized additive model of certain parameters, and/or an analysis of the presented results during other waves of the pandemic.

## Figures and Tables

**Table 1:** Description of cohorts.

Cohort ID	Description	Endpoint
A	ED patients discharged to home and not admitted as inpatient within 72 h	72 h after ED discharge
B	ED patients discharged to home and admitted as inpatient within 72 h	72 h after ED discharge
C	ED patients admitted to hospital in same encounter	Hospital admit
D	Inpatients who do not go to ICU or die in-hospital	Hospital discharge
E	Inpatients who deteriorated (admitted to ICU or died in-hospital)	Deterioration

ED: emergency department; ICU: intensive care unit.

**Table 2:** Description of comparisons performed.

Comparison. ID and Label	Cohorts Compared	Long Description	Values Used for Non-Categorical Data
1 “ED admission”	A	ED patients discharged to home and not admitted as inpatient within 72 h	Last value during ED stay
	B & C	ED patients who were admitted to the hospital within 72 h	
2 “ED bounce-back”	A	ED patients discharged to home and not admitted as inpatient within 72 h	Last value during ED stay
	B	ED patients discharged to home and admitted as inpatient within 72 h	
3 “Inpatient deterioration”	D	Inpatients who do not go to ICU or die in-hospital	Most recent value measured at midpoint of hospital stay
	E	Inpatients who deteriorate (admitted to ICU or die in-hospital)	Most recent value measured X hours prior to deterioration (X = 4, 12)

## Data Availability

Restrictions apply to the availability of these data. Data was obtained from Banner Health and are only available with the appropriate legal and ethical permissions.

## Appendix A

**Table A1:** ED patient characteristics (age, race, ethnicity, sex), ED length of stay, and outcomes. The “Missing” categories were not included in the calculation of proportions or statistical analysis.

Feature		Overall	Discharged to Home and Not Admitted within 72 h (A)	Discharged to Home and Admitted within 72 h (B)	Admitted within Same Encounter (C)	Admitted within 72 h (B&C)	Comparison 1	Comparison 2
							(A vs. B&C)	(A vs. B)
							*p*-Value	*p*-Value
*n*		10,079	4907	157	5015	5172		
Age (years), mean (SD)		51.6 (19.5)	42.4 (16.9)	53.7 (17.2)	60.5 (17.6)	60.3 (17.6)	<0.001	<0.001
Race, *n* (%)	American Indian or Alaska Native	392 (4.6)	185 (4.5)	†	†	207 (4.6)	0.15	0.961
	Black or African American	606 (7.0)	304 (7.5)			302 (6.7)		
	Other	922 (10.7)	460 (11.3)			462 (10.2)		
	White	6692 (77.7)	3128 (76.7)			3564 (78.6)		
	Missing	1467 (-)	-			-		
Ethnicity, *n* (%)	Hispanic or Latino	4623 (49.0)	2526 (55.2)	†	†	2097 (43.1)	< 0.001	0.114
	Not Hispanic or Latino	4816 (51.0)	2051 (44.8)			2765 (56.9)		
	Missing	640 (-)	-			-		
Sex, *n* (%)	Female	5359 (53.2)	2797 (57.0)	†	†	2562 (49.5)	< 0.001	0.206
	Male	4719 (46.8)	2110 (43.0)			2609 (50.5)		
	Missing	1 (-)	-			-		
ED length of stay (h), mean (SD)		7.3 (21.4)	3.9 (6.4)	4.9 (5.0)	10.6 (29.1)	10.5 (28.7)	< 0.001	0.023
Mortality, *n* (%)			-NA-	11 (7.0)	446 (8.9)	457 (8.8)		
ICU admit, *n* (%)			-NA-	27 (17.2)	1081 (21.6)	1108 (21.4)		
Hospital Length of Stay (h), mean (SD)			-NA-	179.4 (152.0)	157.5 (146.9)	158.3 (147.1)		

SD: standard deviation. †: Withheld due to low counts (<100). -NA-: Not Applicable because patients were discharged to home.

**Table A2:** Demographic characteristics of non-critical inpatients with and without deterioration outcomes. The “Missing” categories were not included in the calculation of proportions or statistical analysis. SD: standard deviation.

Feature		Overall	Admitted to ICU or Died in Hospital (E)	Not Admitted to ICU or Died in Hospital (D)	Comparison 3 (D vs. E)*p*-Value
***n***		4766	970	3796	
**Age (years), mean (SD)**		59.8 (18.5)	66.5 (18.3)	58.1 (18.2)	<0.001
**Race, *n* (%)**	**American Indian or Alaska Native**	261 (6.3)	42 (5.0)	219 (6.6)	0.117
	**Black or African American**	267 (6.4)	44 (5.2)	223 (6.7)	
	**Other**	419 (10.0)	86 (10.2)	333 (10.0)	
	**White**	3228 (77.3)	673 (79.6)	2555 (76.7)	
	**Missing**	591 (-)			
**Ethnicity, *n* (%)**	**Hispanic or Latino**	1792 (40.2)	324 (35.9)	1468 (41.3)	0.004
	**Not Hispanic or Latino**	2669 (59.8)	579 (64.1)	2090 (58.7)	
	**Missing**	305 (-)			
**Sex, *n* (%)**	**Female**	2456 (51.5)	420 (43.3)	2036 (53.6)	<0.001
	**Male**	2310 (48.5)	550 (56.7)	1760 (46.4)	
	**Missing**	0 (-)			

## Appendix C

**Table A3:** Multivariate coefficients and *p*-values for Comparison 1 (ED admission).

Feature	Coefficient	*p*-Value
Oxygen support	2.19	<0.001
Age	0.54	<0.001
headache	−0.52	<0.001
Fatigue	0.58	<0.001
Neutrophils count	0.39	<0.001
Anion gap	0.38	<0.001
SpO2	−0.35	<0.001
New or worsening cough	−0.33	<0.001
eGFR	−0.31	<0.001
Lymphocytes %	0.27	<0.001
Respiratory Rate	0.26	<0.001
Temperature (core body)	0.23	<0.001
Calcium	−0.22	<0.001
Platelets: Lymphocytes	0.21	<0.001
ALP	0.21	<0.001
Hgb	−0.19	<0.001
Glucose	0.17	<0.001
Eosinophils %	0.14	<0.001
Systolic BP	−0.13	<0.001

**Table A4:** Multivariate coefficients and *p*-values for Comparison 2 (ED bounce-back).

Feature	Coefficient	*p*-Value
Fatigue	0.59	0.008
Platelet count	−0.39	<0.001
Neutrophils %	0.33	<0.001
Age	0.33	<0.001
Diastolic BP	−0.23	0.009
Anion gap	0.19	0.006
SpO2	−0.19	0.003

## Appendix D

**Table A5:** Multivariate regression coefficients for comparison 3, 12 h prior to the event.

Feature	Coefficient	*p*-Value
Oxygen support	1.68	<0.001
Heart Rate	0.53	<0.001
Respiratory Rate	0.45	<0.001
Neutrophils %	0.43	<0.001
SpO2	−0.38	<0.001
Age	0.36	<0.001
Anion gap	0.31	<0.001
GCS	−0.31	<0.001
Temperature (core body)	0.26	<0.001
Platelet count	−0.22	<0.001
Calcium	−0.18	0.001
BUN:Creatinine	0.16	0.004
Diastolic BP	−0.16	0.004
RDW SD	0.13	0.01
WBC	0.12	<0.01
Bilirubin total	0.12	0.004

**Table A6:** Multivariate regression coefficients for comparison 3, 4 h prior to the event.

Feature	Coefficient	*p*-Value
Oxygen support	1.31	<0.001
Respiratory Rate	0.72	<0.001
SpO2	−0.58	<0.001
Heart Rate	0.50	<0.001
Anion gap	0.45	<0.001
Lymphocytes %	−0.38	<0.001
GCS	−0.34	<0.001
Age	0.34	<0.001
Temperature (core body)	0.26	<0.001
Platelet count	−0.26	<0.001
RDW CV	0.17	<0.001
Systolic BP	−0.16	0.002
Neutrophils:Lymphocytes	0.15	0.006
Lymphocytes count	0.12	0.006

**Table A7:** Ranks and numbers of times covariates appear in the various multivariate models.

Feature	Rank Comparison 1	Rank Comparison 2	Rank Comparison 3: 12 h	Rank Comparison 3: 4 h	Number of Models
SpO2	7	7	5	3	4
Age	2	4	6	8	4
Anion gap	6	6	7	5	4
Oxygen support	1		1	1	3
Platelet count		2	10	10	3
Temperature (core body)	12		9	9	3
Respiratory Rate	11		3	2	3
Lymphocytes %	10			6	2
Diastolic BP		5	13		2
Calcium	13		11		2
Fatigue	4	1			2
GCS			8	7	2
Heart Rate			2	4	2
Systolic BP	19			12	2
Neutrophils %		3	4		2
Bilirubin total			16		1
WBC			15		1
RDW CV				11	1
RDW SD			14		1
BUN:Creatinine			12		1
Neutrophils:Lymphocytes				13	1
Hgb	16				1
Eosinophils %	18				1
Glucose	17				1
ALP	15				1
Platelets: Lymphocytes	14				1
eGFR	9				1
New or worsening cough	8				1
Neutrophils count	5				1
headache	3				1
Lymphocytes count				14	1
